# A country-wide malaria survey in Mozambique. I. *Plasmodium falciparum *infection in children in different epidemiological settings

**DOI:** 10.1186/1475-2875-7-216

**Published:** 2008-10-24

**Authors:** Samuel Mabunda, Sónia Casimiro, Llorenç Quinto, Pedro Alonso

**Affiliations:** 1 National Malaria Control Programme, Maputo, Mozambique; 2 National Institute of Health, Maputo, Mozambique; 3 Centre for International Health, Institut d'Investigacions Biomedicas August Pi i Sunyer (IDIBAPS), Barcelona, Spain; 4 Centro de Investigaçao em Saude da Manhiça, Maputo, Mozambique

## Abstract

**Background:**

Across tropical Africa the bulk of malaria-related morbidity and mortality is particularly high during childhood. Classical malariometric surveys have relied on assessing malaria infection prevalence. The last comprehensive evaluation of the malaria situation in Mozambique was carried out during the 1950s. This study aims to characterize the malaria transmission intensities and to estimate the disease burden that may help guide control programme.

**Methods:**

Between February 2002 and April 2003, a house-to-house survey, was carried out in 24 districts randomly selected. A total of 8,816 children aged below 10 years old were enrolled. Finger prick and blood collection were performed to prepare thick and thin films for malaria parasite species identification, density and haemoglobin concentration. Axillary temperature was also measured. Prevalence of infection, parasite density and anaemia were estimated for age groups category in each region/stratum. Comparisons between proportions were made using Chi-square test or Fisher exact. Relationship between age groups, region/stratum and parasite prevalence, density was determined using linear regression. All survey mean estimations were adjusted for sampling weights, clustering and stratification.

**Results:**

Malaria parasite prevalence was 58.9% (5.190/8.816), the majority of blood smears 52.4% (4,616/8,816) were due to *Plasmodium falciparum *and geometric mean parasite density was 1,211 parasites/μl (95% CI, 1,141 – 1.286). *G*ametocytes prevalence, only for *P. falciparum *was 5.6% (518/8,816). The burden was highest in the northern regions and in the coastal stratum. Parasite infection and geometric mean parasite density peaked during the second year of life and thereafter decreased with increasing age. Mean haemoglobin concentrations was 9.9 g/dl (95% CI 9.5 – 10.2). Anaemia prevalence was 69.8% (6.257/8.816) and among anaemic children 11.5% (743/6.257) were severely anaemic. Anaemia rose dramatically during the first year of life to peak among children in the 12 – 23 months age group. Highest levels of anaemia were recorded in both northern and central-northern regions 77.9% and 79.4% respectively.

**Conclusion:**

This survey confirms that malaria especially that caused by *P. falciparum*, remains endemic throughout the country. The burden of malaria disease and anaemia-related malaria during childhood constitute a major public health problem and warrant integrated and collaborative interventions towards its control.

## Background

Malaria, especially that caused by *Plasmodium falciparum*, remains one of the most important causes of morbidity and mortality among impoverished communities across endemic regions of sub-Saharan Africa [1-3]. Conservative estimates of the burden of disease claim for more than 300 million clinical episodes and 1–3 million deaths every year, and young children harbour the large and most important portion of this toll [4-7]. Nevertheless, the true magnitude and impact of the disease remain imprecise [8,9].

Traditionally, estimation of the burden of malaria relies primarily on mortality and morbidity data collected by the health system information. In most endemic malaria countries in sub-Saharan Africa, the existing health information systems report merely hospital-based data. Moreover, the health system network do not cover the immense rural areas where the majority of the populations live, hence a substantial number of malaria cases and deaths occurring beyond the formal health system are not reported in either hospital or national vital statistics [10,11].

Recently, data from surveys carried out in several malaria endemic regions have been used for evaluating the patterns of malaria infection endemicity across regions and mapping malaria prevalence to estimate population at risk [12-14]. Moreover, routine assessment of malaria infection prevalence is essential to an effective planning, implementing and monitoring of malaria control interventions [15]. The last comprehensive assessment of malaria prevalence in the country was carried out during the early 1950's.

A countrywide survey was conducted to perform a methodologicaly sound assessment of the epidemiological malaria situation in order to determine the prevalence and intensity of infection among children less than 10 years of age across different ecological settings in the country. This study was part of a routine malaria surveillance conducted by the National Malaria Control Programme – Ministry of Health.

## Materials and methods

### Study area

A country-wide survey was conducted in Mozambique, from February 2002 to April 2003. The country is stretched north-to-south, and thus was stratified into four regions namely northern, centre-northern, central and southern. Each region encompasses three distinct ecological settings distinguished as stratum coastal with altitude below 200 meters above sea level, where the majority of population inhabits; stratum plateau with altitude between 200 and 600 meters above sea level, and highland stratum situated above 600 meters above sea level.

The climate is tropical and humid, commonly influenced by the monsoons from the Indian Ocean and the hot current of the Mozambique Channel. There are predominantly two climatic seasons, one hot and wet from September/October to April/May characterized by tropical rainstorm, high temperatures and high relative humidity. The dry and cold season characterised by windy weather and relatively low temperatures.

The wet season lasts for about six to eight months in the central and northern regions, while in southern region is much shorter between four and six months.

In areas of high altitude the annual average temperature varies between 18°C and 20°C, whilst the coastal stratum in the northern and centre-northern regions, the annual average temperatures varies between 26°C and 28°C, and in the coastal plains of the southern region the annual average temperatures varies between 22°C and 26°C. In the Plateau strata, the average temperatures are much higher, generally above 28°C.

Malaria, predominantly caused by *Plasmodium falciparum*, is endemic. Transmission is perennial with peaks during and after rainy seasons. The most important vectors responsible for transmission belong to the *Anopheles gambiae *complex and the *Anopheles funestus *group.

### Sampling method and study population

A modified two-stage cluster-sampling method was adopted. The primary sampling units were the districts. In total, 24 districts were randomly selected, and 30 cluster units were designed per district. A cluster unit consisted of eight congregated households chosen randomly. In each district the list with estimated number of households for all Administrative Units was produced from the 1997 census data. The number of clusters to be sampled in each Administrative Units was calculated based on the sampling with probability proportional to the population size of the Administrative Unit.

All children aged below ten years of life, living in the selected households, were eligible for the survey. Oral informed consent was obtained and a questionnaire was completed by well-trained team members.

### Clinical and laboratory procedures

In each participating child axillary temperature was measured using electronic thermometer. Blood samples were collected to prepare thick and thin film for parasitological examination. Simultaneously blood samples were used to estimate haemoglobin concentration using the HemoCue System (HemoCue, Anglholm, Sweden).

Fever was defined as an axillary temperature ≥ 37.5°C. Anaemia was defined as haemoglobin concentration value below the age specific level; for children under five years old was 11.0 g/dl and from 5 to 11 years old was 11.5 g/dl. Children presenting with fever and positive for the rapid malaria test, received a treatment dose of chloroquine or alternatively sulphadoxine/pyrimethamine, accordingly to the national policy.

Thick and thin blood films were stained with Giemsa following standard quality-controlled procedure [8] and examined twice for malaria parasites species identification and density by trained microscopists, in the laboratory of the National Institute of Health – Ministry of Health. Cases of discrepancy between the two readings, as well as a random control check of 10% of the slides, were performed by a third microscopist. Parasite densities were obtained counting the number of asexual *P. falciparum *parasites per 500 leukocytes and a final density was calculated using an assumed leukocyte count of 8,000/mm^3^.

### Data management and statistical analysis

Data collected were double-entered using a Data Management for Field Trials (DMFFT2) run over a Windows NT network and cleaned before databases were locked. A data manager performed daily cross-checking routines to compare and correct any discrepancies between the two entries. Discordances detected at this point were recorded in a log file permitting quality control of the data checking process.

Checks for duplicate records, completeness of the databases, consistency and referential integrity were also performed, and the final analysis was conducted using Stata 8.1 (Stata Corporation, College Station, TX, USA). Children were grouped into five age group categories: less than 12 months of age; 12 to 23 months; 24 to 59 months of age; 5 to less than 7 years of age; and 7 to less than 10 years of age.

The summary measures of all categorical variables consisted of means, the percentages, the 95% confidence interval and standard deviations for continuous variables. Accounting for the sample design, there are three factors arising from the design of data collection procedure, namely: Sampling Weights, Clustering and Stratification.

### Sampling weights

Observations were selected through a random process, hence may have different probability of being selected; Clustering – Observations were sampled as a group (clusters), therefore, not independently; and Stratification – The stratum categorization was made in advance and sampling was done independently across each strata. Consequently, strata and regions are statistically independent and therefore can be analysed as such. Adjustments to the weights were done and the estimators obtained were approximately unbiased for all point prevalence estimated.

Therefore, clustering and the stratification of the survey design was considered and estimates of standard errors, valid p-values, and confidence intervals whose true coverage are close to 95% were attained. Additionally to handling Clustering and Stratification effects, the design effects to measure how the survey design affects variance estimates were calculated. Thus, all survey mean estimations were adjusted for sampling weights, stratification and clustering. The design effect "deff" was computed automatically.

The prevalence of *falciparum *malaria infection, fever and malaria parasite infection associated with fever were estimated for each age group category for each region and stratum. Parasite density for each age group is shown as geometric mean parasite density, after log_10 _transformation. Comparisons between proportions (in different age groups) were carried out using the Chi-square test (χ^2 ^test) of Pearson or Fisher exact test if any expected frequency is lower than 5. The relationship between age, stratum or region and fever prevalence, parasite prevalence, parasite density and anaemia was determined using linear regression method.

## Results

### Demographic characteristics of children

Of the 8,816 children enrolled during the survey 47% (4,143/8, 8816) were male and 53% (4,763/8,816) were female. The mean age was 42 months, and the 24–59 months age group was the largest category 39.9% (3,515/8,816)

### Laboratory and clinical findings

Overall, 58.9% (5,190/8,816) of blood smears obtained from participating children were positive for malaria parasites. The summary of some malariometric indicators are illustrated in Table 1. The majority of blood smears, 52.4% (4,616/8,816) exhibited a pure *P. falciparum *infection, 3.6% (321/8,816) were *Plasmodium malariae *and 2.9% (253/8,816) were mixed infections of *P. falciparum *and *P. malariae*. Gametocytes (sexual forms) only for *P. falciparum *were recorded in 5.9 % (518/8,816) of all blood smears. There were no records of infections by other parasite species, namely *Plasmodium ovale *or *Plasmodium vivax*. *P. falciparum *accounted for 88.9% (4,616/5.190) of all malaria parasite infections. The overall geometric mean parasite density only for *P. falciparum *asexual parasites was 1,211 parasites/μl (95% CI, 1,141 – 1.286). Axillary temperature ranged between 35.1°C and 40.5°C (SD 0.67). The mean temperature was 36.7°C (95% CI 36.6°C – 36.9°C). Haemoglobin concentrations ranged from 1.5 to 19.7 g/dl, and overall mean estimation was 9.9 g/dl (95% CI 9.5 – 10.2). The prevalence of anaemia was 69.8% (6.257/8.816) and among anaemic children 11.5% (743/6.257) were severely anaemic.

**Table 1:** Overall distribution of malariometric indicators by age groups among children in Mozambique

	Mean Estimation	<12 m	12–23 m	24–59 m	5 – <7 y	7 – <10 y	p-Value
*P. falciparum* (%)	48.6	42.2	55.4	51.3	48.1	39.3	0.0002
Parasite density (CI 95%)	1,211 (1,141–1,286)	1,671 (1,422–1,963)	1,939 (1,698–2,213)	1,172 (1,072–1,281)	673 (577–785)	650 (538–784)	
Fever (%)	9.4	15.1	13.2	7.1	5.9	6.5	0.0000
Fever & parasites (%)	6.3	10.6	10.3	4.6	3.2	3.3	0.0000
Haemoglobin (CI 95%)	9.9 (9.5–10.2)	9.2 (8.6–9.8)	9.0 (8.7–9.4)	9.9 (9.7–10.3)	10.6 (10.3–10.9)	10.9 (10.7–11.3)	
Anaemia (%)	69.8	81.2	83.6	70.7	53.4	46.5	0.0000
Gametocytes (%)	5.6	6.5	7.2	5.8	3.5	3.3	0.0285
*P. malariae* (%)	3.6	3.2	5.2	3.9	3.1	1.1	0.0172
Mixed infection (%)	2.9	2.6	4.9	3.0	2.6	0.3	0.0003

*P. falciparum *= Asexual forms; Gametocytes = Sexual forms only for *P. falciparum*; Mixed infection = Concomitant presence of *P. falciparum *and *P. malariae*; Parasite Density = Geometric mean parasite density only for *P. falciparum*, after log10 transformation (expressed as asexual parasites/μl; Fever = Axillary temperature ≥ 37.5°C; Fever & Parasites = Fever associated with one or more asexual forms of *P. falciparum *parasites; Anaemia = Haemoglobin < 11 g/dl.

NB.: All point prevalence are mean estimation adjusted for stratification, clustering and design effect.

### Overall mean estimation of malaria parasite prevalence and mean parasite density

**Figure 1:**
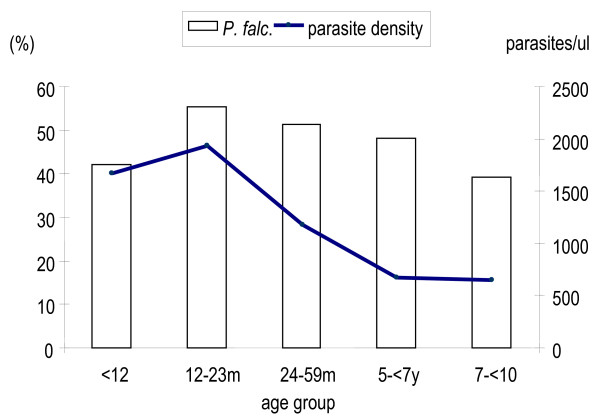
Overall *Plasmodium falciparum *prevalence and mean parasite density among children less than ten years of age in Mozambique.

The study area was markedly endemic for *P. falciparum*, hence attention was limited to *P. falciparum *infections. Overall, prevalence of *P. falciparum *was 52.4% (4,616/8,816) (95% CI, 40.0% – 57.3%). Figure 1, depicts the distribution of malaria infection prevalence among children under ten years old. There was a significant variation between age groups (p = 0.0002). It increased with age from 42.2% in children less than 12 months old to reach a peak of 55.4% among children aged between 12 – 23 months old, and thereafter it decreased progressively to the lowest prevalence of 39.3% among older children in the seven years to less than 10 years old age group. In relation to gender, the proportions of *P. falciparum *infection among boys and girls were not significantly different (p = 0.746).

Overall, mean parasite density increased during the first year of life from 1,671 parasites/μl (95% CI 1,425.21 – 1,961.83), peaking among the children 12 – 23 months age group to 1,939 parasites/μl (95% CI 1,698 – 2,213). The distribution among age groups showed significant differences (p = 0.0001). Despite, the presence of relatively high prevalence of *P. falciparum *parasites among older children, low mean parasite densities were confined to older children. Based on age-specific densities, mean parasite density showed also an age-dependent variation (p = 0.0001), decreasing dramatically with age, as illustrated in Figure 1.

Overal, prevalence of *P. falciparum *gametocytes was 5.6% (95% CI, 3.6% – 7.5%). The highest prevalence of gametocytes (7.2%) was recorded among children in the 12 – 23 months age group. Thereafter, the prevalence of gametocytes decreased with age. Although, there was a significant variation between age groups (p = 0.029), there were no significant differences between boys and girls (p = 0.158).

**Figure 2:**
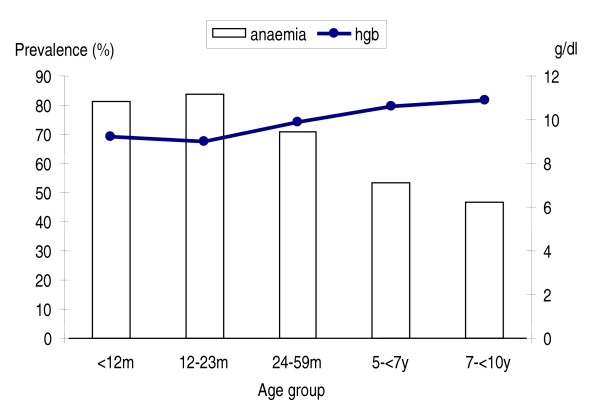
Overall mean haemoglobin concentration and prevalence of anaemia among children less than ten years of age in Mozambique.

*Plasmodium malariae *prevalence was 3.6% (95% CI, 1.3% – 5.9%). It increased from 3.2% during the first 12 months of life, peaking among children in the 12 – 23 months age group, afterwards decreased rapidly with age (Figure 2). Variations on distribution among age groups were statistically significant (p = 0.017), but no significant differences were observed between male and female children (p = 0.435). Overall, prevalence of mixed infection was 2.9% (95% CI, 0.6% – 5.3%). The highest prevalence (4.9%) was recorded among children aged between 12 and 23 months. Rare mixed infection were recorded in older children, and there were no significant differences between boys and girls (p = 0.313).

### Prevalence of fever and fever associated with malaria parasites

Overall, fever prevalence (axillary temperature ≥ 37.5°C) among children was 9.4% (766/8,816). The prevalence of fever peaked among children during the first 12 months of life [15.1% (206/1,517)]. The lowest fever prevalence of 5.9% (67/1,224) was recorded among children in the 5- to 7-year age group. Similarly to malaria parasite density, fever prevalence decreased rapidly with age.

Overall the prevalence of fever associated with *P. falciparum *infections (asexual forms) accounted for 5.7% (498/8,816). The prevalence of malaria infection associated with fever peaked among children during the first year of life and thereafter decreased sharply with increasing age, and the differences among age groups were statistically significant (p < 0.001).

The risk of being febrile increased with increasing parasite density, particularly from parasite density category equal or higher than 5,000 parasites/μl. High *P. falciparum *parasite densities were significantly associated with fever (p < 0.05). According to age group, the risk of fever increased during the first 12 months of life and thereafter decreased significantly with age (p < 0.0001).

Across the study area there were significant regional variations (p < 0.001). The highest prevalence of malaria infection associated with fever was recorded in the northern region and the lowest was recorded in the southern region.

### Overall mean estimation of haemoglobin and prevalence of anaemia

Overall, mean estimation of haemoglobin concentration was 9,9 g/dl (95% CI, 9.5 – 10.2), the prevalence of anaemia using the 11.0 g/dl altitude adjusted race-specific WHO cut off was 69.8% and among anaemic children 11.5% had severe anaemia (haemoglobin less than 5 g/dl).

There was considerable variation in the prevalence of anaemia among age groups (p < 0.0001). In general, all age groups had low haemoglobin concentration, and consequently high levels of anaemia prevalence. Children during the second year of life had the lowest haemoglobin concentration, thereafter, haemoglobin concentration increased with increased age as is illustrated Figure 2. There were no significant differences between male and female children in mean haemoglobin concentration or prevalence of anaemia at any level (p = 0.554).

Approximately half of anaemic children had *P. falciparum *parasites infection associated, while *P. malariae *infections were recorded in 4.5%, and mixed infections by *P. falciparum *and *P. malariae *accounted for 3.6% among anaemic children.

### Regional variations

**Figure 3:**
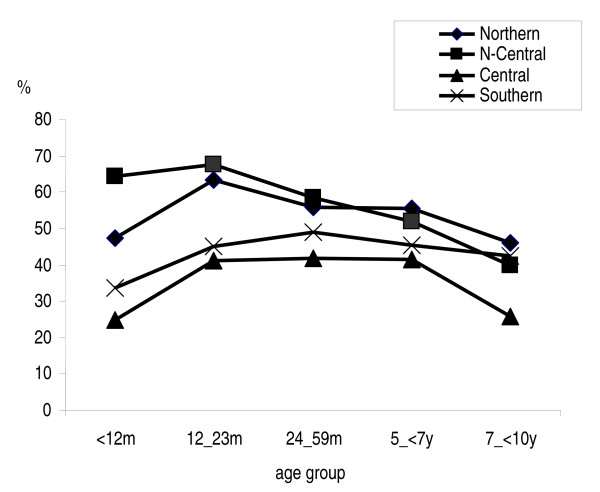
Overall prevalence of *Plasmodium falciparum *infection among children less than ten years of age, across regions in Mozambique.

Overall, the prevalence of malaria infection showed variations throughout various regions in the country, decreasing from north-to-south (Figure 3). The highest overall prevalence of *P. falciparum *infection was recorded in both northern and the central-northern regions 54.8% (1,313/2,387) and 58.7% (992/1,929), respectively. Whereas the lowest overall prevalence of 36.8% (1,180/2930) and 44.6% (613/1,570) corresponding to central and southern regions.

In the northern and central-northern regions, the prevalence of *P. falciparum *infection, increased during the first 12 months of life from 47.5% and 64.3%, peaking to 63.3% and 67.7%, respectively among the 12 – 23 months age group children, and thereafter decreased progressively with age. Only in the central-northern region, significant differences in age group variations were observed (p = 0.042). Within the same age groups, the peak observed of 41.2% and 44.9% across central and southern regions respectively, was much lower. Thereafter, the prevalence decreased slightly with very little variation among older children. Differences in the distribution between age groups were observed only for the central region (p < 0.0001).

**Figure 4:**
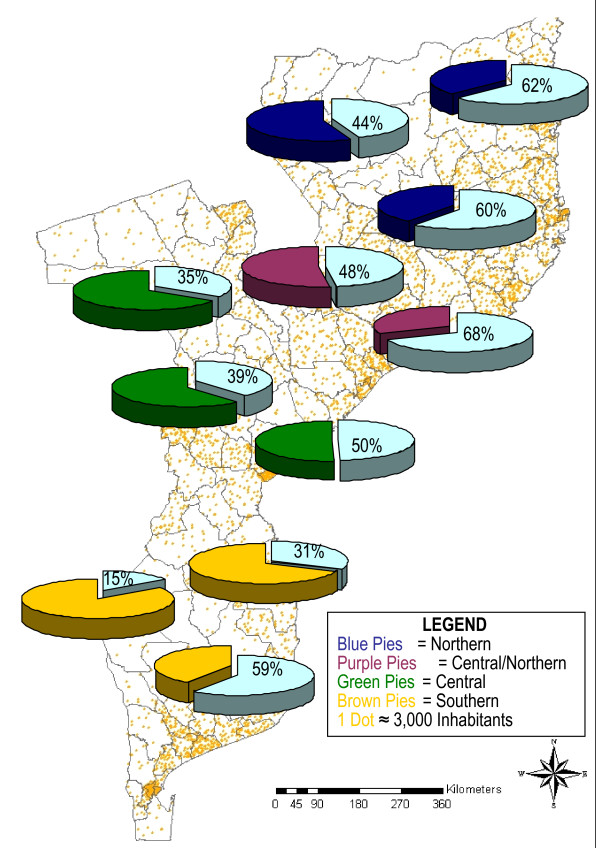
Mean estimation point prevalence of *Plasmodium falciparum *infection among children aged bellow ten years old across different epidemiological settings in Mozambique.

Across strata, there was a significant decrease of *P. falciparum *infection prevalence from the low lands of the coastal stratum to the highland stratum in both central (p = 0.02) and southern (p = 0.004) regions, while the variations observed across strata in the northern region and in the northern-central region were not statistically significant (Figure 4).

Similarly, the overall mean parasite density showed a considerable regional variation. High mean parasite density of 2,058 parasites/μl (95% CI, 1,836–2,306) was recorded in children across the central-northern region, and young children below 12 months of age harbour the maximum load of parasite density 3,494 parasites/μl, (95% CI 2,641–4,621). Comparatively, low mean parasite density of 891 parasites/μl (95% CI, 799–994) was recorded among children in central region. The overall mean parasite density in the northern and southern regions was 1,077 parasites/μl (95% CI, 965–1,200) and 1,193 parasites/μl (95% CI, 1,025 – 1,388), respectively.

Generally, in both northern regions, mean parasite density peaked during the first 12 months of age, while in central and southern regions the peak was recorded later among children in the 12 – 23 months of life. Nonetheless, in all regions, parasite mean densities were markedly age-dependent, and decreased sharply with age (p < 0.0001).

There was a significant regional variation on the prevalence of *P. falciparum *gametocytes (p = 0.002). Nevertheless, no significant differences were observed across strata of all regions (p = 0.55). Overall the central and central-northern regions recorded the highest prevalence of gametocytes 7.3% (131/2,387) and 8.2% (136/1,929), respectively. The lowest prevalence was recorded in the southern region 2.0% (29/1,570).

Regions with low prevalence showed an erratic distribution of gametocytes, according to age groups. However, across regions with high gametocytes prevalence, it increased among children less than 12 months of age, and peaking among children in the 12–23 months of age. In the central-northern region the peak was observed earlier during the first 12 months of age. Thereafter, the prevalence of gametocytes decreased considerably with age (p = 0.029).

The prevalence of *P. malariae *parasites was relatively low in the study area, accounting for 3.6% (518/8,816) of malaria infections. Overall, the prevalence of *P. malariae *infection showed significant regional variations (p = 0.013). The highest was recorded in the central-northern regions 7.4% (124/1,929). While in the central region was recorded the lowest prevalence 1.4% (59/2,930). Across strata, there was a significant decrease of *P. malariae *infection prevalence from 6.8% in the low lands of the coastal stratum to 1.7% in the highland stratum (p = 0.026).

In general, the prevalence of *P. malariae *infection varied significantly with age (p = 0.02). The peak of infection was recorded among younger children aged between 12 – 23 months old, and thereafter decreased gradually with age. No difference was observed between males and females.

Episodes of mixed infections were observed predominantly in the central-northern region 7.0% (117/1,929) and in the northern region 3.1% (82/2,387). The occurrence of mixed infections in both central 0.9% (41/2,930) and southern 0.8% (13/1,570), regions were negligible.

Variations in the distribution of mixed infection showed significant differences among age groups. The peak of mixed infections was recorded among children in the 24–59 months age group.

### Prevalence of fever and fever associated with malaria parasites infection

Overall, high fever prevalence were recorded in the northern and central-northern regions 12.8% (287/2,387) and 10.8% (187/1,929), while in the central and southern region were recorded the lowest fever prevalence of 6.9% (185/2,930) and 7.2% (107/1,570) respectively. Although the prevalence of fever showed significant regional variations (p = 0.019), declining from north to south following the same pattern of malaria parasites infection distribution, there were no significant differences within strata across the regions. In all regions fever prevalence decreased with age, though a slight increase were observed among children aged 7 years and above, except in the central-northern region.

Fever associated with malaria parasites was markedly high in the northern and central-northern regions; 9.1% (199/2,387) and 8.4% (139/1,929), respectively. The central region registered the lowest prevalence of fever associated with parasites 6.9% (101/2,930). In spite of high variations of the proportions of fever associated with malaria parasites, the differences across regions were not statistically significant (p = 0.108).

### Overall mean haemoglobin and prevalence of anaemia

Mean haemoglobin concentration showed insignificant differences between regions. In general, there was some degree of variations on mean haemoglobin concentration across regions. The northern and central-northern regions recorded mean haemoglobin of 9.4 g/dl (95% CI, 8.3 – 10.4) and 9.4 g/dl (95% CI, 8.5 – 10.3), respectively. A slightly higher mean haemoglobin concentration of 10.2 g/dl (95% CI, 9.8 – 10.7) and 10.4 g/dl (95% CI, 9.8 – 10.9) were recorded in the central and in the southern regions, respectively.

According to age groups, mean haemoglobin concentration, increased significantly with age only within the central (p < 0.05) and southern (p < 0.005) regions. In the northern regions also increased, but without significant differences among age groups.

**Figure 5:**
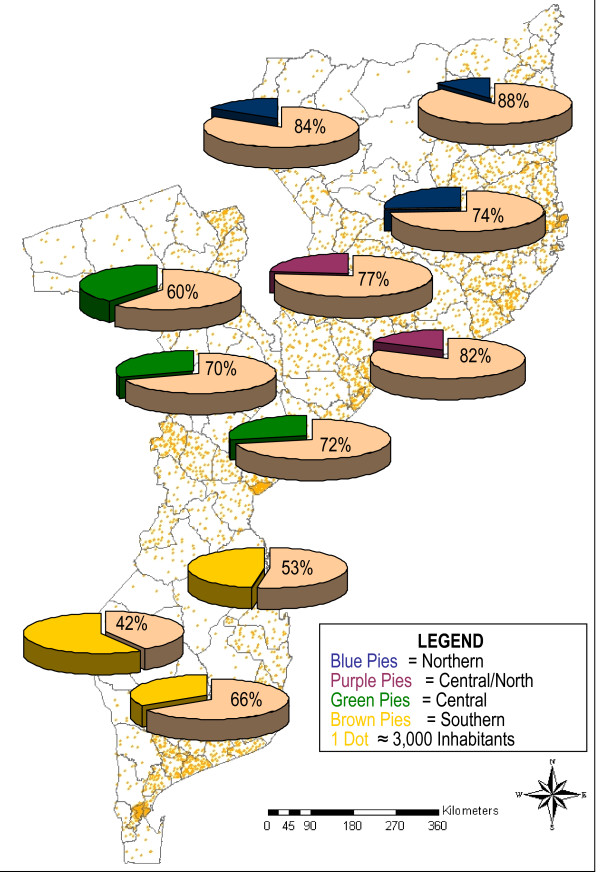
Mean estimation point prevalence of anaemia among children aged bellow ten years old, across different epidemiological settings in Mozambique.

Overall, the highest levels of anaemia prevalence were recorded in the northern and central-northern regions 77.9% and 79.4% respectively (Figure 5). Although significant regional differences on the levels of anaemia (p = 0.0002), coincidently, in all regions the prevalence of anaemia rose dramatically among children less than 12 months of age to peak in children in the 12 – 23 months age group. There were not differences on anaemia prevalence across strata, except within the northern region where the coastal stratum had high proportion of anaemia and significantly different from other strata (p = 0.049).

The levels of severe anaemia were higher among children in the northern and central northern regions 2.2% and 1.6%. The lowest prevalence of severe anaemia was recorded in the southern region (0.3%), and the differences observed between regions were statistically significant (p = 0.031). Overall, the peak was recorded among children in the 12 – 23 months age group. However, the northern region recorded the highest peak (3.9%) of severe anaemia prevalence among children aged below 12 months. Unlike prevalence of anaemia, severe anaemia decreased sharply with age. In the southern region there were no records of severe anaemia among children aged 5 years and above.

Comparison among anaemic and non-anaemic subjects in all regions, revealed a significant association between prevalence of anaemia and malaria parasite infections. Among anaemic children 54.0% had *P. falciparum *parasites, while among non-anaemic 37.6% had malaria infection associated (p = 0.011).

## Discussion

Planning and monitoring of malaria control programmes requires good quality data that can define disease burden across geographical and ecological areas in a country. A nationwide survey was performed to assess the malaria disease burden, by estimating the prevalence and intensity of *Plasmodium *infections and anaemia in children less than 10 years of age across different ecological settings in Mozambique.

Overall, the prevalence of malaria infection and parasite density among children was markedly age-dependent, peaking during the first year of life, and thereafter declining with increasing age, as described previously in other malaria endemic areas across tropical Africa [16,17], which traditionally has been interpreted as a slow build up of anti-parasite immunity. Therefore, as children grow up exposed to repeated infective mosquito bites, they acquire an ability to limit the occurrence of high density infections, and to reduce the occurrence of fever [17].

This study highlighted variations on the intensity of malaria infection across the country, decreasing from northern to southern regions and from low lands in the coastal and plateau strata to the highlands stratum. In general the heterogeneity in malaria transmission and its intensity within regions could be explained by the regional variations on the amount of rainfall, average air temperatures, humidity and also on the human population distribution. These findings corroborate with the results from the first and only attempt to evaluating the malaria situation nationwide conducted by Soeiro *et al *during the 1950s, which showed highest prevalence of malaria infections in the centre-northern region and in the coastal areas of the country [18]. Despite, malaria control efforts (i.e. insecticide indoor residual spraying restricted to major urban areas and case management) that have subsequently been implemented throughout the country, may have reduced malaria infection transmission, 50 years later the malaria situation is almost similar. It could be argued that the 1970s and 1980's decades where characterized by the emergence and rapid spread of malaria parasite strains resistant to first line anti-malarials, vector resistance and generalized collapse of public health services, nevertheless, in the absence of appropriate and adequate malaria control interventions, most likely malaria infection rates may have reverted to natural pre-operational levels.

Data from a longitudinal field research with a strong focus on epidemiology and entomology conducted during the 1970's, in Garki have demonstrated that malaria in tropical Africa is characterized by high levels of transmission and the pattern of malaria endemicity is maintained by highly effective vectors. Regardless of high coverage rates of residual spraying with an effective insecticide against the mosquito vectors, combined with mass drug administration at high frequency and coverage, reduction on malaria transmission to low levels was observed, but interruption could not be achieved [19]. Equally, in some tropical regions, the global campaign to eradicate malaria produced enormous reductions in the burden of disease. However, malaria cases rebounded dramatically after cessation of control activities [20]. Similar results were reported from repeated cross-sectional survey data collected from one historical trial of indoor residual spraying against malaria vector in two contiguous districts in Tanzania-Kenya the Pare-Taveta project [21].

This study confirmed that malaria especially that caused by *P. falciparum*, continues to be a major scourge throughout the country and therefore represents a large public health problem. Young children bear the brunt of the infection and this implies exposure to intense malaria transmission. Parasite rates in children may well guide to a classification of the malaria endemicity, as described by Metselaar and van Thiel [20]. The method has been routinely used as a "border line" marker of malaria endemicity levels across sub-Saharan Africa [20,22]. In general, along the coastline and in the flat terrains in the northern regions, malaria transmission can be categorized as hyperendemic. While in the highlands strata across central and southern regions can be categorized as mesoendemic.

Moreover, results of this study strongly support the finding that *P. falciparum *malaria plays a key role in the burden of anaemia [24,17,26]. The results have shown high levels of anaemia, particularly among younger children. These findings are in agreement with results from studies in other malaria-endemic areas [23,25]. Similarly, the finding that parasitized children carried a strikingly high burden of anaemia compared to non-parasitized children corroborate with results from other community surveys carried out in malaria-endemic areas [25-27]. Nevertheless, other risk factors such as nutritional deficiencies mainly iron and folate deficiencies, intestinal parasitic diseases such as bilharzias, intestinal helmintics have been associated with high prevalence of anaemia during childhood [28,23]. Anaemia impacts on survival and cognitive development during childhood. Poor intellectual performance and decreased physical exercise have been associated with low haemoglobin concentration in children [29]. Given that anaemia is a key risk factor for survival and cognitive development, its control should become a public health priority for the country.

## Conclusion

The burden of malaria disease and anaemia-related malaria during childhood constitutes a major public health problem. According to 1997 population census projection, if we apply the age regional and stratum specific prevalence rates for malaria infection and anaemia, at a position in time in the country we can estimate absolute numbers accordingly. Thus, approximately 2.6 million of children less than 10 years of age are expected to harbour *P. falciparum *malaria parasites and approximately 3.8 million are expected to be anaemic. The majority of these children inhabit the coastal and plateau strata in the northern regions.

Integrated and collaborative malaria control interventions are warranted. Intermittent preventive treatment, insecticide treated nets, mass de-worming, iron, and vitamin A supplementation programmes have already proven to be cost-effective interventions, particularly in areas lacking adequate health care services. In future malaria vaccines may also contribute to improving control and prevention efforts. Moreover, estimations of the disease burden caused by malaria are essential for planning cost-effectively malaria control interventions, monitoring and advocacy.

## Competing interests

The authors declare that they have no competing interests.

## Authors' contributions

SJAM, made a substantial contribution on conception and design of the study, co-ordinated and supervised data collection in all regions, executed all statistical analysis and wrote the manuscript. SLRC, co-ordinated and supervised data collection in the central-northern region, revised the manuscript. LQ made a major contribution on study design and helped with statistical analysis. PA gave a major contribution on conception and study design and helped to draft and critically revised the manuscript. All authors read and approved the final manuscript.
